# Synthesis of Micheliolide Derivatives and Their Activities against AML Progenitor Cells

**DOI:** 10.3390/molecules18055980

**Published:** 2013-05-21

**Authors:** Wei-Wei Ma, Qian-Qian Shi, Ya-Hui Ding, Jing Long, Quan Zhang, Yue Chen

**Affiliations:** 1 College of Pharmacy, the State Key Laboratory of Elemento-Organic Chemistry, and Tianjin Key Laboratory of Molecular Drug Research, Nankai University, Tianjin 300071, China; E-Mails: mww23@126.com (W.-W.M.); jingtufei@163.com (J.L.); 2 College of Food Engineering and Biotechnology, Tianjin University of Science and Technology, Tianjin 300457, China; E-Mail: shiqian2634@126.com; 3 Accendatech Co., Ltd, Tianjin 300384, China; E-Mail: chuanshuo2200@sina.com

**Keywords:** leukemia progenitor cell, micheliolide, KG-1a, deriviative, sythesis

## Abstract

Micheliolide (MCL) derivatives with etherification or esterification of the hydroxyl group at the C4 position were synthesized and evaluated for their activities against different acute myelogenous leukemia (AML) cell lines. These derivatives demonstrated comparable activities against AML cell lines HL-60 and doxorubicin resistant cell line HL-60/A. As to multi-drug resistant AML progenitor cells KG-1a, MCL and some of its derivatives maintained significant activities, and only 1.1–2.7 fold activity reductions were observed when compared with the activities against HL-60, while doxorubicin showed 20-fold activity reduction. Our study demonstrated that the C4 hydroxyl group of MCL might not only be a suitable position for structural modifications, but also be a starting point for the design of appropriate molecular probes to explore the specific targets in the progenitor cell line KG-1a.

## 1. Introduction

Leukemia is one of the most aggressive adult cancers, as well as the most prevalent childhood cancer. Leukemia is a cancer of the hematological system and can be divided into a diversity of unique malignancies based on the onset of the disease as well as the specific cell lineages involved [1]. Acute myelogenous leukemia (AML) is a malignant disease characterized by an aberrant accumulation of immature myeloid hematopoietic cells. Multi-drug resistance and relapse are the main difficulties in the treatment of most AML patients. Recent studies have indicated that the drug resistance and relapse of AML arises from a rare population of leukemic stem cells (LSCs), and new therapeutics targeting LSC are urgently needed [2,3,4]. KG-1a cells are a type of short-term CD34+ hematopoietic progenitor cell line, and contain leukemia stem-like cells characterized by the CD34+CD38− biomaker [5]; in some cases, the leukemic stem-like cells comprise about 50% of the total KG-1a cells [6]. KG-1a cells exhibit high p-glycoprotein-mediated drug efflux capacity and a high level of anthracycline resistance [2]. Thus, KG-1a cells present a relevant cellular model for leukemic stem cells research.

**Figure 1:**
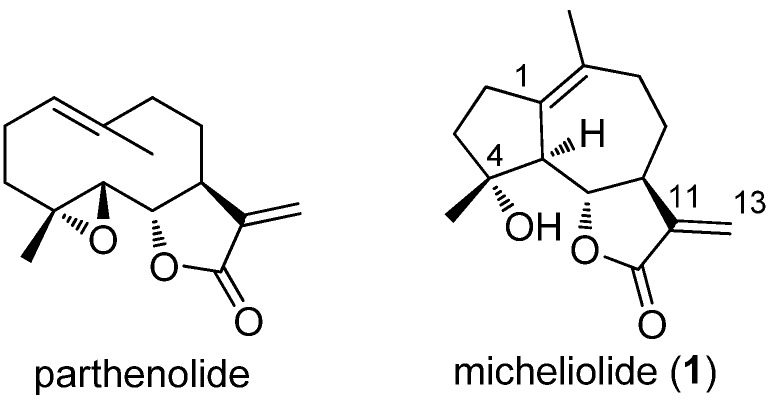
Structure of parthenolide (PTL) and micheliolide (MCL, **1**).

Parthenolide (PTL, Figure 1), a naturally occurring germacrane sesquiterpene lactone, was identified as a promising agent targeting AML stem cell populations [7,8]. DMAPT, the water-soluble dimethylamino Michael adduct of PTL, has entered clinical trials in the United Kingdom for the treatment of AML, ALL, and CLL [9,10]. Recently, we reported that guaianolide sesquiterpene lactones could selectively inhibit AML stem and progenitor cells, and micheliolide (MCL, **1**, Figure 1) was identified as the lead compound for reducing the proportion of AML stem cells (CD34+CD38−) in primary AML cells. Moreover, the dimethylamino Michael adduct of MCL, DMAMCL, demonstrated remarkable therapeutic efficacy in the AML NOD/SCID mice models, and very low acute toxicity in mice [11]. In view of the high therapeutic potential of MCL and DMAMCL, we were interested in further investigation of the structure-activity relationships of MCL. MCL, a guaianolide sesquiterpene lactone [12] isolated from *Michelia compressa* [13] and *Michelia champaca* [14], was also prepared by semisynthesis from parthenolide in 90% yield [15]. Thus, with MCL as the starting material, we synthesized a series of MCL derivatives and investigated their biological activities as potential anti-AML progenitor agents.

## 2. Results and Discussion

### 2.1. Chemistry

**Scheme 1:**
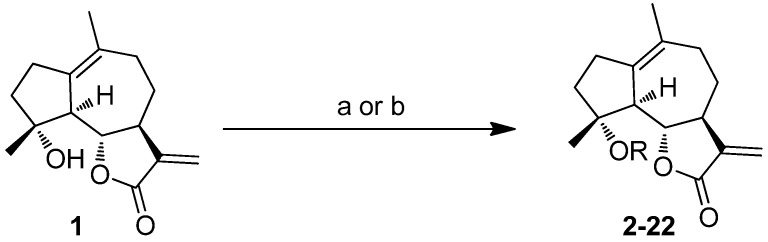
Synthesis of compounds **2–22**.

**Scheme 2:**
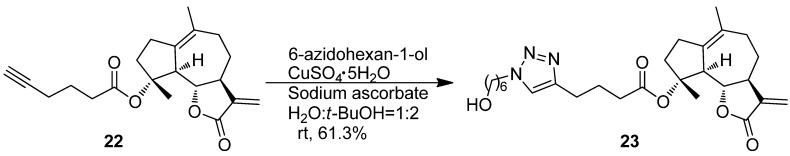
Synthesis of compound **23**.

It has been proposed that a parent molecule could be made more cytotoxic by increasing the lipophilic character and/or by adding alkylating groups [16,17]. The presence of the free hydroxyl group at C4 position allowed us to prepare ether or ester derivatives of compound **1**. As shown in Scheme 1, compound **1** was treated with CH_3_I, benzyl bromide, or CH_3_OCH_2_Cl to provide etherified derivatives **2**, **3** and **4** respectively. Reaction of compound **1** with different acyl chloride in the presence of 4-dimethylaminopyridine (DMAP) or NaH gave compounds **5–10**, **13–16**, and **18–22** in low to moderate yields (14%–78%). Treatment of compound **1** with carboxylic acids in the presence of *N*,*N'*-dicyclohexylcarbodiimide (DCC) and DMAP afforded compounds **11** and **12**. Synthesis of compound **17** was achieved via Sc(OTf)_3_ catalyzed reaction of compound **1** and 5-bromopentanoic acid in the presence of diisopropylcarbodiimide (DIPC) and DMAP [18]. The 1,3-dipolar cycloaddition with compound **22** and 6-azidohexan-1-ol in the presence of CuSO_4_·5H_2_O and sodium ascorbate afforded compound **23** (Scheme 2).

*Reagents and conditions*: (a) for compound 2: CH_3_I, NaH, THF, rt, 36%; for compound 3: CH_3_OCH_2_Cl, NaH, THF, rt, 64%; for compound 4: benzyl bromide, NaH, THF, rt, 40%; (b) for compounds **5**, **6**, **9**, **10**, **13**, and **18–22**: RCl, Et_3_N, DMAP, CH_2_Cl_2_, rt, 14%–78%; for compounds 7, 8, and 14–16: RCl, NaH, THF, rt, 21%–56%; for compounds **11** and **12**: RCOOH, EDCI, DMAP, CH_2_Cl_2_, rt, 48%–53%; for compound **17**: RCOOH, Sc(OTf)_3_, DIPC, DMAP, CH_2_Cl_2_, rt, 26%.

### 2.2. Activities against AML Cell Lines

This series of MCL derivatives was then tested for activities against cultured AML cell line HL-60 and doxorubicin-resistant cell line HL-60/A. Doxorubicin (DOX), an anti-AML drug in clinical application) was applied as positive control [11]. The results are summarized in Table 1.

Doxorubicin demonstrated over 100-fold reduction against HL-60/A (IC_50_ = 6.7 μM) *vs.* HL-60 (IC_50_ = 0.05 μM). The parent compound **1** showed comparable inhibitory potency to HL-60/A (6.2 μM) and HL-60 (IC_50_ = 5.5 μM) [11]. For the etherified compound **2**, masking of the C4-OH with a methyl group also resulted in comparable activities against HL-60/A (IC_50_ = 10.2 μM) and HL-60 (IC_50_ = 9.9 μM). Replacement of the methyl moiety with a more bulky substituent (compound **3**) slightly reduced the anti-AML activity, and potent inhibitory activities against HL-60 and HL-60/A were found to be retained in the methoxymethyl derivative (compound **4**) with IC_50_ values of 3.5 μM and 6.2 μM against HL-60 and HL-60/A, respectively.

**Table 1:** Inhibitory effects of compounds **1–23** on HL-60, HL-60/A, and KG-1a cell lines ^a^. 
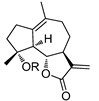

Compounds	R	IC_50_^b^ (µM)			Times ^f^
		HL-60 ^c^	HL-60/A ^d^	KG-1a ^e^	
DOX ^g^	-	0.05 ± 0.01	6.7 ± 1.1	1.0 ± 0.3	20
**1** (MCL)	H	5.5 ± 1.4 ^h^	6.2 ± 2.2 ^h^	13.4 ± 1.0	2.4
**2** ^h^	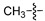	9.9 ± 0.9	10.2 ± 0.1	-	-
**3**	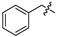	16.7 ± 0.8	18.9 ± 4.6	-	-
**4**	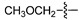	3.5 ± 0.6	6.2 ± 0.4	-	-
**5**	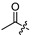	12.0 ± 3.2	8.3 ± 2.3	-	-
**6**	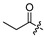	10.0 ± 0.6	15.6 ± 0.1	-	-
**7**	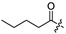	11.8 ± 1.4	14.2 ± 2.2	15.9 ± 0.9	1.3
**8**	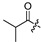	13.7 ± 1.7	22.0 ± 1.4	-	-
**9**	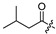	10.1 ± 2.2	12.5 ± 0.3	11.5 ± 1.4	1.1
**10**	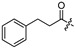	15.7 ± 1.7	15.1 ± 4.2	-	-
**11**	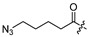	16.1 ± 3.1	29.7 ± 5.9	-	-
**12**	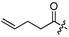	12.4 ± 0.2	18.0 ± 1.0	-	-
**13**	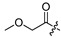	7.2 ± 2.7	15.7 ± 3.0	19.4 ± 4.2	2.7
**14**	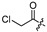	16.1 ± 1.9	20.3 ± 5.7	-	-
**15**	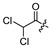	18.0 ± 3.4	20.0 ± 3.2	-	-
**16**	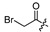	2.8 ± 0.9	4.2 ± 0.2	7.5 ± 0.9	2.7
**17**	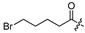	13.7 ± 2.6	16.7 ± 1.0	-	-
**18**	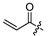	7.4 ± 1.6	8.5 ± 1.8	10.4 ± 2.3	1.4
**19**	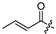	12.6 ± 0.2	11.7 ± 2.2	-	-
**20**	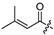	13.1 ± 1.2	17.6 ± 4.6	-	-
**21**	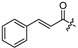	15.1 ± 1.9	14.7 ± 1.9	-	-
**22**	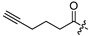	8.1 ± 1.4	10.8 ± 1.9	10.5 ± 0.6	1.3
**23**	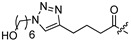	16.4 ± 4.3	29.0 ± 1.4	-	-

^a^ All values are the mean of three independent experiments; ^b^ IC_50_: 50% cytotoxic concentration; ^c^ HL-60: cultured AML cell line; ^d^ HL-60/A: doxorubicin-resistant cell line; ^e^ KG-1a: AML progenitor cell line; ^f^ Ratio of the IC_50_ value for KG1a compared to the IC_50_ value for HL-60; ^g^ DOX: doxorubicin, a clinically popular anti-AML agent used as a positive control; ^h^ Data from reference [11].

The esterified subseries of compounds **5–13**, **24**, **23** have different substitution patterns on the C4 position. As shown in Table 1, these compounds generally exhibited slightly reduced or comparable activities against HL-60 (IC_50_ = 7.2–16.1 μM) and HL-60/A (IC_50_ = 8.3–29.7 μM). This result indicates that a range of substituents with different lipophilic, electronic, and steric characters is tolerated at the C4 position of compound 1. The most potent compound in this subseries was compound 13, with IC_50_ values of 7.2 μM and 15.7 μM against HL-60 and HL-60/A, respectively, which was comparable to that of compound 1.

As to the haloacylated subseries of compounds **14–17**, compound **16** exhibited high activities against HL-60 (IC_50_ = 2.8 μM) and HL-60/A (IC_50_ = 4.2 μM), suggesting additional alkylating groups appear to enhance anti-AML activity (cf. compound **16** to compound **1**). However, compounds **14**, **15**, and **17** showed decreased activity, which indicates that there is an optimum property, number, and position for the substitution of α halogen atoms. When a conjugated ester group (*i.e.*, compounds **18–21**) was added to the compound 1 to supply additional Michael receptors, the anti-AML activities were mainly retained. Moreover, the gradation of activities exhibited by compounds **18–21** demonstrated that along with the increment of the steric hindrance of the conjugated ester, the activities against HL-60 were declining.

The relatively potent compounds **7**, **9**, **13**, **16**, **18**, and **22** were further screened to obtain their inhibitory activities against AML KG-1a progenitor cells (Table 1). Compared with MCL, these compounds maintained comparable activities against the KG-1a cell line (IC_50_ = 7.5–19.4 μM). The most potent compound was compound **16** (IC_50_ = 7.5 μM), which was slightly more potent than MCL (IC_50_ = 13.4 μM). Moreover, MCL and derivatives **7**, **9**, **13**, **16**, **18**, and **22** maintained significant activities, and only 1.1–2.7 fold activity reductions were observed when compared with their activities against HL-60, while doxorubicin demonstrated over 20-fold reduction against KG-1a (IC_50_ = 1.0 μM) *vs.* HL-60 (IC_50_ = 0.05 μM).

## 3. Experimental

### 3.1. General

The starting material micheliolide (MCL, **1**) was obtained from Accendatech Co., Ltd. (Tianjin, China). The solvents used were purified and dried according to common procedures. ^1^H-NMR (400 MHz) and ^13^C-NMR spectra (100 MHz) were obtained on a Bruker AV 400 instrument using CDCl_3_ as the solvent. Chemical shifts are reported in parts per million (ppm) relative to either a tetramethylsilane internal standard or solvent signals. Data are reported as follows: chemical shift, multiplicity (s = singlet, d = doublet, t = triplet, q = quartet, br. = broad, m = multiplet), coupling constants and integration. High-resolution mass spectra were recorded using an Agilent 6520 Q-TOF LC/MS instrument by the ESI-FTICR technique.

### 3.2. Product Synthesis and Characterization

#### *General Procedure for the Synthesis of Compounds* **5**, **6**, **9**, **10**, **13**, and **18–22**

To a solution of MCL (24.8 mg, 0.1 mmol), Et_3_N (121.2 mg, 1.2 mmol), and DMAP (12.2 mg, 0.1 mmol) in anhydrous CH_2_Cl_2_ (1 mL) was added appropriate acyl chloride (1.0 mmol) at 0 °C. The resulting mixture was stirred at room temperature until the starting material disappeared on the TLC. The reaction mixture was diluted with water (2 mL), extracted with CH_2_Cl_2_ (5 mL × 2). The organic layer was successively washed with saturated citric acid (5 mL × 3), NaHCO_3_ (5 mL × 3), and brine (5 mL × 3), and then dried over anhydrous Na_2_SO_4_, concentrated under reduced pressure to give a crude residue, which was purified by silica gel column chromatography to afford the product.

*(3aS,9R,9aS,9bS)-6,9-Dimethyl-3-methylene-2-oxo-2,3,3a,4,5,7,8,9,9a,9b-decahydroazuleno*[4,5-b]*furan-9-yl acetate* (**5**): yield 63%, white amorphous powder; ^1^H-NMR δ 6.13 (1H, d, *J* = 3.1 Hz), 5.40 (1H, d, *J* = 2.8 Hz), 3.72 (1H, t, *J* = 10.2 Hz), 3.08 (1H, d, *J* = 10.1 Hz), 2.65–2.59 (1H, m), 2.41–2.35 (2H, m), 2.21–2.13 (4H, m), 2.04–2.00 (1H, m), 1.97 (3H, s), 1.91–1.83 (1H, m), 1.65 (3H, s), 1.48 (3H, s); ^13^C-NMR δ 169.6, 169.3, 138.4, 130.6, 129.4, 117.8, 87.7, 82.0, 55.6, 49.1, 35.4, 33.9, 29.4, 24.9, 23.2, 21.5, 17.8; HRMS (ESI) for C_17_H_23_O_4_, calcd 291.1596, found 291.1593.

*(3aS,9R,9aS,9bS)-6,9-Dimethyl-3-methylene-2-oxo-2,3,3a,4,5,7,8,9,9a,9b-decahydroazuleno*[4,5-b]*furan-9-yl propionate* (**6**): yield 70%, white amorphous powder; ^1^H-NMR δ 6.20 (1H, d, *J* = 3.1 Hz), 5.47 (1H,d, *J* = 2.8 Hz), 3.79 (1H, t, *J* = 10.1 Hz), 3.13 (1H, d, *J* = 10.2 Hz), 2.71–2.66 (1H, m), 2.49–2.42 (2H, m), 2.38–2.30 (2H, m), 2.28–2.26 (4H, m), 2.11–2.08 (1H, dd, *J* = 13.8, 1.8 Hz), 1.97–1.89 (1H, m), 1.72 (3H, s), 1.57 (6H, d, *J* = 9.0 Hz); ^13^C-NMR δ 173.8, 170.2, 139.5, 131.5, 130.4, 118.6, 88.4, 83.0, 56.6, 50.1, 36.5, 34.9, 30.4, 28.7, 25.9, 24.1, 18.8, 9.1; HRMS (ESI) for C_18_H_28_NO_4_, calcd 322.2018, found 322.2013.

*(3aS,9R,9aS,9bS)-6,9-Dimethyl-3-methylene-2-oxo-2,3,3a,4,5,7,8,9,9a,9b-decahydroazuleno*[4,5-b]*furan-9-yl 3-methylbutanoate* (**9**): yield 48%, white amorphous powder; ^1^H-NMR δ 6.19 (1H, d, *J* = 3.2 Hz), 5.46 (1H, d, *J* = 2.9 Hz), 3.78 (1H, t, *J* = 10.1 Hz), 3.13 (1H, d, *J* = 10.1 Hz), 2.71–2.66 (1H, m), 2.50–2.42 (2H, m), 2.28–2.26 (3H, m), 2.20–2.07 (4H, m), 1.97–1.89 (1H, m), 1.72–1.69 (4H, m), 1.56 (3H, s), 0.97 (6H, d, *J* = 5.9 Hz); ^13^C-NMR δ 172.7, 170.2, 139.5, 131.6, 130.5, 118.7, 88.5, 83.0, 56.8, 50.2, 44.6, 36.6, 35.0, 30.5, 25.9, 25.8, 24.2, 22.4, 22.3, 18.8; HRMS (ESI) for C_20_H_32_NO_4_, calcd 350.2331, found 350.2326.

*(3aS,9R,9aS,9bS)-6,9-Dimethyl-3-methylene-2-oxo-2,3,3a,4,5,7,8,9,9a,9b-decahydroazuleno*[4,5-b]*furan-9-yl 3-phenylpropanoate* (**10**): yield 24%, white amorphous powder; ^1^H-NMR δ 7.32-7.18 (5H, m), 6.22 (1H, d, *J* = 3.3 Hz), 5.49 (1H, d, *J* = 3.0 Hz), 3.80 (1H, t, *J* = 10.1 Hz), 3.12 (1H, d, *J* = 10.1 Hz), 2.99 (2H, t, *J* = 7.9 Hz), 2.73–2.59 (3H, m), 2.49–2.43 (2H, m), 2.30–2.28 (3H, m), 2.14–2.10 (1H, dd, *J* = 13.8, 2.3 Hz), 1.96–1.87 (1H, m), 1.74 (3H, s), 1.65 (1H, s), 1.56 (3H, s); ^13^C-NMR δ 172.3, 170.2, 140.8, 139.5, 131.6, 130.4, 128.4, 126.0, 118.7, 88.8, 83.0, 56.7, 50.1, 36.8, 36.5, 35.0, 31.0, 30.5, 26.0, 24.2, 18.8; HRMS (ESI) for C_24_H_32_NO_4_, calcd 398.2331, found 398.2328.

*(3aS,9R,9aS,9bS)-6,9-Dimethyl-3-methylene-2-oxo-2,3,3a,4,5,7,8,9,9a,9b-decahydroazuleno*[4,5-b]*furan-9-yl 2-methoxyacetate* (**13**): yield 14%, white amorphous powder; ^1^H-NMR δ 6.20 (1H, d, *J* = 2.9 Hz), 5.47 (1H, d, *J* = 2.4 Hz), 4.07–3.95 (2H, q, *J* = 16.4Hz), 3.79 (1H, t, *J* = 10.1 Hz), 3.47 (3H, s), 3.19 (1H, d, J = 10.1 Hz), 2.71–2.66 (1H, m), 2.51–2.44 (2H, m), 2.27 (3H, s), 2.11–2.08 (1H, dd, *J* = 12.5, 0.7 Hz), 2.04–1.95 (1H, m), 1.72 (3H, s), 1.63 (1H, s), 1.58 (3H, s); ^13^C-NMR δ 170.2, 169.7, 139.3, 131.9, 130.0, 118.9, 89.6, 82.9, 70.3, 59.3, 56.4, 50.0, 36.4, 35.0, 30.4, 25.9, 24.2, 18.9; HRMS (ESI) for C_18_H_28_NO_5_, calcd 338.1967, found 338.1960.

*(3aS,9R,9aS,9bS)-6,9-Dimethyl-3-methylene-2-oxo-2,3,3a,4,5,7,8,9,9a,9b-decahydroazuleno*[4,5-b]*furan-9-yl acrylate* (**18**): yield 78%, white amorphous powder; ^1^H-NMR δ 6.47–6.37 (1H, dd, *J* = 17.3, 1.3Hz), 6.21 (1H, d, *J* = 3.3 Hz), 6.13–6.06 (1H, m), 5.80–5.77 (1H, dd, *J* = 10.3, 1.3 Hz), 5.48 (1H, d, *J* = 3.1 Hz), 3.82 (1H, t, *J* = 10.1 Hz), 3.15 (1H, d, *J* = 10.1 Hz), 2.73–2.67 (1H, m), 2.58–2.44 (2H, m), 2.29–2.27 (3H, m), 2.12–2.08 (1H, dd, J = 13.7, 2.3 Hz), 2.00–1.92 (1H, m), 1.73 (3H, s), 1.59 (4H, s); ^13^C-NMR δ 170.3, 165.5, 139.4, 131.7, 130.2, 130.1, 130.0, 118.8, 88.8, 83.0, 57.1, 50.1, 36.5, 35.0, 30.5, 25.9, 24.2, 18.6; HRMS (ESI) for C_18_H_26_NO_4_, calcd 320.1862, found 320.1859.

*(E)-(3aS,9R,9aS,9bS)-6,9-Dimethyl-3-methylene-2-oxo-2,3,3a,4,5,7,8,9,9a,9b-decahydroazuleno*[4,5-b]*furan-9-yl but-2-enoate* (**19**): yield 14%, white amorphous powder; ^1^H-NMR δ 6.22 (1H, d, *J* = 3.3 Hz), 5.49 (1H, d, *J* = 3.0 Hz), 5.20 (1H, d, *J* = 5.0 Hz), 5.16 (1H, s), 3.81 (1H, t, *J* = 10.1 Hz), 3.16 (1H, d, *J* = 8.8 Hz), 3.12–3.09 (2H, m), 2.74–2.68 (1H, m), 2.51–2.44 (2H, m), 2.30–2.28 (4H, m), 2.14–2.10 (1H, dd, J = 13.7, 2.1 Hz), 2.00–1.92 (1H, m), 1.88–1.86 (1H, dd, *J* = 6.9, 1.3 Hz), 1.74 (3H, s), 1.59 (1H, s), 1.58 (3H, s); ^13^C-NMR δ 171.0, 170.2, 139.5, 131.7, 130.7, 130.3, 118.7, 118.2, 89.0, 83.0, 56.7, 50.1, 40.2, 36.5, 35.0, 30.5, 25.9, 24.2, 18.8; HRMS (ESI) for C_19_H_25_O_4_, calcd 317.1753, found 317.1747.

*(3aS,9R,9aS,9bS)-6,9-Dimethyl-3-methylene-2-oxo-2,3,3a,4,5,7,8,9,9a,9b-decahydroazuleno*[4,5-b]*furan-9-yl 3-methylbut-2-enoate* (**20**): yield 27%, white amorphous powder; ^1^H-NMR δ 6.20 (1H, d, *J* = 3.2 Hz), 5.68 (1H, s), 5.47 (1H, d, *J* = 2.8 Hz), 3.81 (1H, t, *J* = 10.2 Hz), 3.15 (1H, d, *J* = 10.0 Hz), 2.73–2.67 (1H, m), 2.57–2.43 (2H, m), 2.28–2.27 (3H, m), 2.15 (3H, s), 2.12–2.08 (1H, dd, *J* = 13.9, 2.1 Hz), 2.00–1.92 (2H, m), 1.87 (3H, s), 1.73 (3H, s), 1.59 (3H, s); ^13^C-NMR δ 170.3, 166.2, 155.4, 139.5, 131.5, 130.6, 118.7, 117.7, 88.2, 83.1, 57.0, 50.3, 36.8, 34.9, 30.6, 27.4, 25.9, 24.2, 20.1, 18.9; HRMS (ESI) for C_20_H_27_O_4_, calcd 331.1909, found 331.1903.

*(3aS,9R,9aS,9bS)-6,9-Dimethyl-3-methylene-2-oxo-2,3,3a,4,5,7,8,9,9a,9b-decahydroazuleno*[4,5-b]*furan-9-yl cinnamate* (**21**): yield 23%, white amorphous powder; ^1^H-NMR δ 7.76 (1H, d, *J* = 16.0 Hz), 7.59–7.57 (2H, m), 7.39 (3H, d, *J* = 5.0 Hz), 6.45 (1H, d, *J* = 16.0 Hz), 6.24 (1H, d, *J* = 3.1 Hz), 5.51 (1H, d, *J* = 2.7 Hz), 3.88 (1H, t, *J* = 10.2 Hz), 3.20 (1H, d, *J* = 10.0 Hz), 2.78–2.73 (1H, m), 2.65–2.61 (1H, m), 2.55–2.48 (1H, m), 2.31 (3H, s), 2.16 (1H, d, *J* = 13.5 Hz), 2.07–1.98 (1H, q), 1.75 (3H, s), 1.63 (4H, s); ^13^C-NMR δ 170.4, 166.4, 144.5, 139.5, 134.7, 131.6, 130.0, 129.9, 128.8, 128.2, 119.7, 118.8, 88.7, 83.1, 57.3, 50.0, 36.7, 35.0, 30.6, 26.0, 24.2, 18.6; HRMS (ESI) for C_24_H_30_NO_4_, calcd 396.2175, found 396.2167.

*(3aS,9R,9aS,9bS)-6,9-Dimethyl-3-methylene-2-oxo-2,3,3a,4,5,7,8,9,9a,9b-decahydroazuleno*[4,5-b]*furan-9-yl hex-5-ynoate* (**22**): yield 65%, white amorphous powder; ^1^H-NMR δ 6.21 (1H, d, *J* = 3.1 Hz), 5.49 (1H, d, *J* = 2.7 Hz), 3.82 (1H, t, *J* = 10.0 Hz), 3.15 (1H, d, *J* = 9.3 Hz), 2.91–2.82 (3H, m), 2.14–2.10 (1H, m), 2.04-2.01 (3H, m), 1.97–1.87 (8H, m), 1.83–1.75 (6H, m), 1.63 (1H, s); ^13^C-NMR δ 172.4, 170.2, 139.4, 131.7, 130.2, 118.8, 88.8, 83.6, 83.0, 69.0, 56.7, 50.1, 36.5, 34.9, 34.1, 30.5, 25.9, 24.2, 23.7, 18.8, 17.8; HRMS (ESI) for C_21_H_30_NO_4_, calcd 396.2175, found 360.2172.

#### *General Procedure for the Synthesis of Compounds* **7**, **8**, and **14–16**

To a mixture of MCL (99.2 mg, 0.4 mmol), NaH (20 mmol), and THF (2 mL) was added appropriate acyl chloride (12 mmol) at 0 °C. The resulting mixture was stirred at room temperature until the starting material disappeared on the TLC. The reaction mixture was diluted with water (3 mL), extracted with ethyl acetate (5 mL × 2). The organic layer was successively washed with saturated citric acid (8 mL × 3), NaHCO_3_ (8 mL × 3), and brine (8 mL × 3), and then dried over anhydrous Na_2_SO_4_, concentrated under reduced pressure to give a crude residue, which was purified by silica gel column chromatography to afford the product.

*(3aS,9R,9aS,9bS)-6,9-Dimethyl-3-methylene-2-oxo-2,3,3a,4,5,7,8,9,9a,9b-decahydroazuleno*[4,5-b]*furan-9-yl pentanoate* (**7**): yield 38%, white amorphous powder; ^1^H-NMR δ 6.19 (1H, d, *J* = 3.1 Hz), 5.47 (1H, d, *J* = 2.9 Hz), 3.78 (1H, t, *J* = 10.1 Hz), 3.13 (1H, d, *J* = 10.0 Hz), 2.72–2.66 (1H, m), 2.49–2.42 (2H, m), 2.37–2.31 (1H, m), 2.30–2.26 (4H, m), 2.12–2.08 (1H, dd, *J* = 13.7, 2.2 Hz), 1.97–1.88 (1H, m), 1.75 (3H, s), 1.64-1.56 (2H, m), 1.55 (3H, s), 1.40–1.31 (3H, m), 0.92 (3H, t, *J* = 7.3 Hz); ^13^C-NMR δ 173.4, 170.2, 139.5, 131.6, 130.4, 118.7, 88.5, 83.0, 56.8, 50.2, 36.5, 35.2, 34.9, 30.5, 27.1, 25.9, 24.1, 22.2, 18.8, 13.8; HRMS (ESI) for C_20_H_29_O_4_, calcd 333.2066, found 333.2061.

*(3aS,9R,9aS,9bS)-6,9-Dimethyl-3-methylene-2-oxo-2,3,3a,4,5,7,8,9,9a,9b-decahydroazuleno*[4,5-b]*furan-9-yl isobutyrate* (**8**): yield 27%, white amorphous powder; ^1^H-NMR δ 6.21 (1H, d, *J* = 3.0 Hz), 5.48 (1H, d, *J* = 2.8 Hz), 3.81 (1H, t, *J* = 10.1 Hz), 3.15 (1H, d, *J* = 10.3 Hz), 2.74–2.68 (1H, m), 2.57–2.44 (3H, m), 2.30–2.29 (3H, m), 2.14–2.10 (1H, dd, *J* = 13.7, 2.1 Hz), 1.98–1.90 (1H, m), 1.74 (3H, s), 1.66 (1H, s), 1.56 (3H, s), 1.21-1.17 (6H, m); ^13^C-NMR δ 176.6, 170.2, 139.6, 131.5, 130.4, 118.6, 88.2, 83.0, 56.8, 50.1, 36.5, 35.0, 34.7, 30.5, 26.0, 24.2, 19.0, 18.9, 18.7; HRMS (ESI) for C_19_H_30_NO_4_, calcd 336.2175, found 336.2172.

*(3aS,9R,9aS,9bS)-6,9-Dimethyl-3-methylene-2-oxo-2,3,3a,4,5,7,8,9,9a,9b-decahydroazuleno*[4,5-b]*furan-9-yl 2-chloroacetate* (**14**): yield 56%, white amorphous powder; ^1^H-NMR δ 6.23 (1H, d, *J* = 3.3 Hz), 5.50 (1H, d, *J* = 3.1 Hz), 4.14–4.05 (2H, m), 3.81 (1H, t, *J* = 10.1 Hz), 3.20 (1H, d, *J* = 10.0 Hz), 2.74–2.68 (1H, m), 2.54–2.47 (2H, m), 2.30–2.29 (3H, m), 2.15–2.11 (1H, dd, *J* = 13.8, 2.3 Hz), 2.07–1.97 (1H, m), 1.75 (3H, s), 1.62 (4H, s); ^13^C-NMR δ 170.1, 166.4, 139.2, 132.1, 129.8, 119.0, 90.8, 82.8, 56.4, 50.1, 42.0, 36.3, 35.0, 30.3, 25.9, 24.2, 18.8; HRMS (ESI) for C_17_H_25_ClNO_4_, calcd 342.1472, found 342.1467.

*(3aS,9R,9aS,9bS)-6,9-Dimethyl-3-methylene-2-oxo-2,3,3a,4,5,7,8,9,9a,9b-decahydroazuleno*[4,5-b]*furan-9-yl 2,2-dichloroacetate* (**15**): yield 21%, white amorphous powder; ^1^H-NMR δ 6.23 (1H, d, *J* = 3.3 Hz), 5.96 (1H, s), 5.51 (1H, d, *J* = 3.0 Hz), 3.81 (1H, t, *J* = 10.1 Hz), 3.21 (1H, d, *J* = 10.1 Hz), 2.76–2.70 (1H, m), 2.56–2.48 (2H, m), 2.30 (3H, s), 2.15–2.11 (1H, dd, *J* = 13.8, 2.3 Hz), 2.07–1.99 (1H, m), 1.75 (3H, s), 1.66 (4H, s); ^13^C-NMR δ 169.9, 163.4, 139.2, 132.4, 129.5, 119.0, 92.3, 82.5, 65.2, 56.5, 50.1, 36.0, 34.9, 30.2, 25.9, 24.1, 18.6; HRMS (ESI) for C_17_H_24_Cl_2_NO_4_, calcd 376.1082, found 376.1078.

*(3aS,9R,9aS,9bS)-6,9-Dimethyl-3-methylene-2-oxo-2,3,3a,4,5,7,8,9,9a,9b-decahydroazuleno*[4,5-b]*furan-9-yl 2-bromoacetate* (**16**): yield 46%, white amorphous powder; ^1^H-NMR δ 6.22 (1H, d, *J* = 3.3 Hz), 5.49 (1H, d, *J* = 3.0 Hz), 3.86 (2H, s), 3.80 (1H, t, *J* = 10.1 Hz), 3.18 (1H, d, *J* = 9.9 Hz), 2.74–2.68 (1H, m), 2.53–2.46 (2H, m), 2.30–2.29 (3H, m), 2.14–2.10 (1H, dd, *J* = 13.8, 2.3 Hz), 2.06–1.94 (1H, m), 1.74 (3H, s), 1.71 (1H, s), 1.60 (3H, s); ^13^C-NMR δ 170.1, 166.3, 139.3, 132.1, 129.9, 118.9, 90.8, 82.8, 56.5, 50.1, 36.2, 34.9, 30.3, 27.7, 25.9, 24.2, 18.7; HRMS (ESI) for C_17_H_25_BrNO_4_, calcd 386.0967, found 386.0963.

#### *General Procedure for the Synthesis of Compounds* **11** *and* **12**

A mixture of MCL (99.2 mg, 0.4 mmol), appropriate carboxylic acid (4 mmol), EDCI (2.4 g, 12.5 mmol), DMAP (488 mg, 4 mmol), and CH_2_Cl_2_ (10 mL) was refluxed until the starting material disappeared on the TLC. The reaction mixture was diluted with water (15 mL), and then extracted with CH_2_Cl_2_ (10 mL × 2). The organic layer was successively washed with saturated citric acid (10 mL × 3), NaHCO_3_ (10 mL × 3), and brine (10 mL × 2), and then dried over anhydrous Na_2_SO_4_, concentrated under reduced pressure to give a crude residue, which was purified by silica gel column chromatography to afford the product.

*(3aS,9R,9aS,9bS)-6,9-Dimethyl-3-methylene-2-oxo-2,3,3a,4,5,7,8,9,9a,9b-decahydroazuleno*[4,5-b]*furan-9-yl 5-azidopentanoate* (**11**): yield 48%, white amorphous powder; ^1^H-NMR δ 6.22 (1H, d, *J* = 3.3 Hz), 5.49 (1H, d, *J* = 3.0 Hz), 3.81 (1H, t, *J* = 10.2 Hz), 3.37–3.33 (2H, m), 3.16 (1H, d, *J* = 10.1 Hz), 2.74–2.68 (1H, m), 2.51–2.45 (2H, m), 2.41–2.33 (2H, m), 2.31–2.29 (3H, m), 2.14–2.10 (1H, dd, *J* = 13.7, 2.2 Hz), 2.00–1.92 (1H, m), 1.74 (3H, s), 1.73–1.66 (4H, m), 1.62 (1H, s), 1.57 (3H, s); ^13^C-NMR δ 171.5, 169.2, 138.4, 130.7, 129.2, 117.7, 87.7, 82.0, 55.7, 50.1, 49.1, 35.5, 34.0, 33.9, 29.4, 27.2, 24.9, 23.1, 21.2, 17.8; HRMS (ESI) for C_20_H_28_N_3_O_4_, calcd 374.2080, found 374.2077.

*(3aS,9R,9aS,9bS)-6,9-Dimethyl-3-methylene-2-oxo-2,3,3a,4,5,7,8,9,9a,9b-decahydroazuleno*[4,5-b]*furan-9-yl pent-4-enoate* (**12**): yield 53%, white amorphous powder; ^1^H-NMR (400 MHz, CDCl_3_) δ 6.18 (1H, d, *J* = 3.2 Hz), 5.87–5.80 (1H, m), 5.46 (1H, d, *J* = 3.0 Hz), 5.09 (1H, d, *J* = 16.9 Hz), 5.00 (1H, d, *J* = 9.6 Hz), 3.80–3.74 (1H, m), 3.12 (1H, d, *J* = 7.4 Hz), 2.68 (1H, s), 2.45–2.40 (3H, m), 2.37 (4H, s), 2.25 (3H, s), 2.11–2.07 (1H, m), 1,95–1.89 (1H, m), 1.71 (3H, s), 1.54 (3H, d, *J* = 3.8 Hz); ^13^C-NMR (100 MHz, CDCl_3_) δ 172.4, 170.2, 139.5, 136.9, 131.6, 130.3, 118.7, 115.3, 88.7, 82.9, 56.7, 50.1, 36.5, 35.0, 34.6, 30.4, 29.0, 25.9, 24.2, 18.8; HRMS (ESI) for C_20_H_27_O_4_, calcd 331.1909, found 331.1910.

#### *Synthesis of* *(3aS,9R,9aS,9bS)-6,9-Dimethyl-3-methylene-2-oxo-2,3,3a,4,5,7,8,9,9a,9b-decahydroazuleno*[4,5-b]*furan-9-yl 5-bromopentanoate* (**17**)

A mixture of MCL (99.2 mg, 0.4 mmol), 5-bromopentanoic acid (216.0 mg, 1.2 mmol), Sc(OTf)_3_ (118.1 mg, 0.24 mmol), DMAP (293.2 mg, 2.4 mmol), and CH_2_Cl_2_ (10 mL) was stirred at -8 °C for 30 min. To the resulting mixture was added DIPC (302.9 mg, 2.4 mmol). The reaction mixture was stirred at room temperature for 2 h, diluted with water (10 mL), and extracted with CH_2_Cl_2_ (10 mL × 2). The organic layer was successively washed with saturated citric acid (10 mL × 3), NaHCO_3_ (10 mL × 3), and brine (10 mL × 2), and then dried over anhydrous Na_2_SO_4_, concentrated under reduced pressure to give a crude residue, which was purified by silica gel column chromatography to afford compound 17 (42.8 mg, yield 25.8%). ^1^H-NMR δ 6.22 (1H, d, *J* = 3.2 Hz), 5.49 (1H, d, *J* = 2.8 Hz), 3.81 (1H, t, *J* = 10.2 Hz), 3.47 (2H, t, *J* = 6.5 Hz), 3.15 (1H, d, *J* = 10.0 Hz), 2.73–2.68 (1H, m), 2.51–2.44 (2H, m), 2.40–2.32 (2H, m), 2.30 (2H, d, *J* = 6.4 Hz), 2.14–2.10 (1H, dd, *J* = 13.7, 1.9 Hz), 1.99–1.91 (3H, m), 1.83-1.78 (2H, m), 1.74 (3H, s), 1.60 (3H, s), 1.57 (2H, s); ^13^C-NMR δ 172.5, 170.2, 139.4, 131.7, 130.2, 118.8, 88.75, 83.0, 56.7, 50.1, 36.5, 35.0, 34.5, 33.5, 32.0, 30.5, 25.9, 24.2, 23.6, 18.8; HRMS (ESI) for C_20_H_31_BrNO_4_, calcd 428.1436, found 428.1437.

#### *Synthesis of* *(3aS,9R,9aS,9bS)-6,9-Dimethyl-3-methylene-2-oxo-2,3,3a,4,5,7,8,9,9a,9b-decahydroazuleno*[4,5-b]*furan-9-yl 4-(1-(hydroxymethyl)-1H-1,2,3-triazol-4-yl)butanoate* (**23**)

To a solution of compound **22** (200 mg, 0.58 mmol), 6-azidohexan-1-ol (125.4 mg, 0.88 mmol), sodium ascorbate (463 mg, 2.3 mmol), copper sulfate (146 mg, 0.58 mmol) in mixed solvent (*tert*-butyl alcohol/water = 1:1) 3 mL under N_2_ atmosphere. The resulting mixture was stirred for 2 h at room temperature. The reaction mixture was poured into ice water (5 mL) and extracted with ethyl acetate (10 mL × 3). The combined organic layer was washed with brine (10 mL × 2), dried over anhydrous sodium sulfate, concentrated under reduced pressure to give a crude residue, which was purified with chromatography to afford compound **23** (120 mg, yield 61%). ^1^H-NMR δ 7.52 (1H, s), 6.20 (1H, d, *J* = 3.3 Hz), 5.49 (1H, d, *J* = 3.0 Hz), 4.34 (2H, t, *J* = 7.1 Hz), 3.81 (1H, t, *J* = 10.2 Hz), 3.63 (2H, t, *J* = 6.4 Hz), 3.14 (1H, d, *J* = 10.1 Hz), 2.81–2.76 (2H, m), 2.74–2.67 (1H, m), 2.51–2.43 (2H, m), 2.39–2.31 (2H, m), 2.29–2.27 (4H, m), 2.13–2.09 (1H, dd, *J* = 13.8, 2.3 Hz), 2.05–2.00 (3H, m), 1.97–1.88 (5H, m), 1.72 (4H, s), 1.60–1.53 (6H, m); ^13^C-NMR δ 172.6, 170.2, 147.1, 139.4, 131.7, 130.0, 121.3, 118.8, 88.6, 83.1, 62.2, 56.7, 50.0, 49.9, 36.4, 34.9, 34.7, 32.3, 30.4, 30.1, 26.1, 25.8, 25.1, 24.8, 24.7, 24.1, 18.8; HRMS (ESI) for C_27_H_40_N_3_O_5_, calcd 486.2968, found 486.2968.

### 3.3. Biological assay for Activity of Compounds **1–23**

The cell viability assay was carried out using the well documented MTT method. All the tested cells were cultured with drugs for 72 hours before adding the MTT reagent. All the experiments were carried out as triplicated and we tested every compound for three times.

## 4. Conclusions

In summary, a series of MCL derivatives **2–23** were synthesized and assayed for their activities against the cultured AML cell line HL-60 and doxorubicin-resistant cell line HL-60/A. Compounds **7**, **9**, **13**, **16**, **18**, and **22** were selected to test their inhibitory activity against AML KG-1a progenitor cells. Our investigation demonstrated that simple modifications of hydroxyl at the C4 position of MCL can maintain comparable activities against regular AML cell lines HL-60 and HL-60/A, and the progenitor cell line KG-1a. Based on the above results, the following conclusions could be made: (a) suitable etherification of hydroxyl group at the C4 position was found to retain anti-AML activities; (b) acylation of the hydroxyl group at the C4 position generally maintained activities against HL-60, HL-60/A, and KG-1a cell lines; (c) additional alkylating groups appear to enhance anti-AML activity; (d) the steric effects in the introduced conjugated ester groups play a role to the anti-AML activities.

Most importantly, KG-1a is a multi-drug resistant (MDR) cell lines, and many drugs demonstrated significant lower activities against KG-1a [19,20,21]. For example, doxorubicin showed 20-fold reduced activity against KG-1a *vs.* against HL-60 (Table 1), while our selected compounds **7**, **9**, **13**, **16**, **18**, and **22** maintained significant activities against KG-1a (only 1.1–2.7 fold reduction). Moreover, MCL can selectively inhibit AML stem and progenitor cells [11], but the mechanisms responsible for the effects of MCL are still unclear. On the basis of our established structure-activity relationships, we may conclude that the hydroxyl group at C4 of MCL might be a suitable position for the design and synthesis of appropriate molecular probes to explore the specific targets of KG-1a progenitor cells and stem cells. Synthesis and application of molecular probes are in progress in our laboratory, and the results will be reported in due course.
